# Supplementary material for: Single-Mode Control and Individual Nanoparticle Detection in the Ultraviolet Region Based on Boron Nitride Microdisk with Whispering Gallery Mode
Source: Nanomaterials (Basel). 2024 Mar 11;14(6):501. doi: 10.3390/nano14060501 (PMC10975174; doi:10.3390/nano14060501)
Supplement: Supplementary file 1 [file nanomaterials-14-00501-s001.zip › nanomaterials-2876448-supplementary.pdf]

Supplementary Materials

S1

In this part ,we simulated a single 2 μm microdisk at 301 nm to achieve a position-independent accurate detection for particle with a size of 138 nm. There is a 299 nm high-order mode near the main peak 301 nm (see Fig. 3 (b) in the paper). Due to particle scattering loss, the line width of the main peak and high-order mode will all increase, and the existence of high-order mode will destroy the shape symmetry of the main peak of 301 nm and cause detection errors. In contrast, there is no high-order mode near 309 nm, which avoids this situation. In addition, the Q factor of 301 nm is 3229, and which of 309 nm is 2158 (see Fig. 4 (a) in the paper). Based on the evanescent field sensing, the evanescent field of the mode with lower Q factor is more likely to interact with the surrounding particle and has higher detection sensitivity. Therefore, we chose to use 309 nm as the detection wavelength.

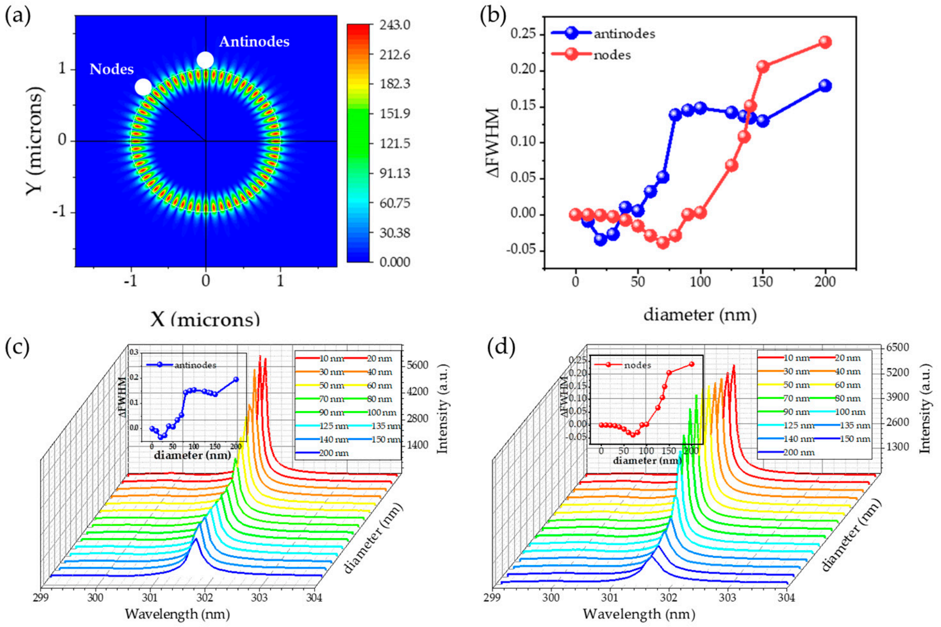

Figure S1. (a) is a schematic diagram of the single PS nanosphere detection at 301 nm with a 2μm disk. (b) is a variation of FWHM (ΔFWHM) as a function of nanospheres with different sizes located at the antinodes and the nodes at 301 nm. (c) and (d) represent the resonant mode variation with different PS nanoparticle sizes at the antinodes and nodes at 301 nm. The inset shows the trend of the ΔFWHM as a function of nanospheres diameter.

S2

We additionally simulated the double-disks coupled particle detection model in Fig. S2. In the double-disks coupling model for single mode, the 2μm disk with the excitation field has a stronger electric field strength, so we placed the probed particle at the antinodes and nodes of the 2μm microdisk while monitoring the amount of ΔFWHM of the main peak at 301 nm. With the increase of particle size, the ΔFWHM at the nodes hardly changes, while there is an obvious trend at the antinodes , as shown in Fig.S2(b). In the coupling, the symmetry of the electric field distributed at the edge of the 2μm microdisk is destroyed, and the field strength distribution located at the nodes moves closer to the center of the microdisk, which affects the size of the evanent field and then limits the particle sensing ability at the nodes. There is a large difference in the ΔFWHM between the antinodes and the nodes, and there is no intersection point. In actual detection situations, we cannot determine whether the particle's position is at the antinodes or the nodes, so it is not possible to judge the accurate information about particle size via this detection method.

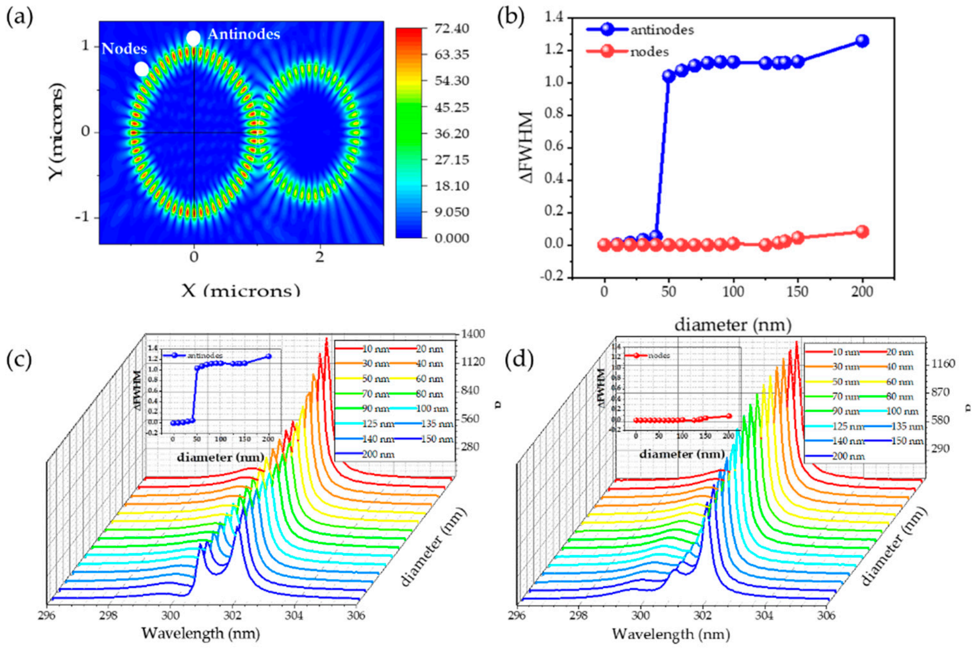

Figure S2. (a) is a schematic diagram of the single PS nanosphere detection at 301 nm with 2μm and 1.6μm coupled disks. (b) is a variation of FWHM (ΔFWHM) as a function of nanospheres with different sizes located at the antinodes and the nodes at 301 nm. (c) and (d) represent the resonant mode variation with different PS nanoparticle sizes at the antinodes and nodes at 301 nm. The inset shows the trend of the ΔFWHM as a function of nanospheres diameter.
